# Using age-specific mortality of HIV infected persons to predict Anti-Retroviral Treatment need: a comparative analysis of data from five African population-based cohort studies

**DOI:** 10.1111/j.1365-3156.2011.02943.x

**Published:** 2012-07-30

**Authors:** Basia Żaba, Ivan Kasamba, Sian Floyd, Raphael Isingo, Kobus Herbst, Till Bärnighausen, Simon Gregson, Constance Nyamukapa, Ndoliwe Kayuni, Jim Todd, Milly Marston, Alison Wringe

**Affiliations:** 1 London School of Hygiene and Tropical MedicineLondon, UK; 2 Medical Research Council/Uganda Virus Research InstituteEntebbe, Uganda; 3 National Institute for Medical ResearchMwanza, Tanzania; 4 Africa Centre for Health and Population Studies, Somkhele, University of KwaZulu NatalKwaZulu Natal, South Africa; 5 Harvard School of Public HealthBoston, MA, USA; 6 Imperial CollegeLondon, UK; 7 Biomedical Research and Training InstituteHarare, Zimbabwe; 8 Karonga Prevention StudyChilumba, Malawi

**Keywords:** antiretroviral therapy, lifetable models, AIDS, HIV, cohort studies, mortality, Africa

## Abstract

**Objectives:**

To present a simple method for estimating population-level anti-retroviral therapy (ART) need that does not rely on knowledge of past HIV incidence.

**Methods:**

A new approach to estimating ART need is developed based on calculating age-specific proportions of HIV-infected adults expected to die within a fixed number of years in the absence of treatment. Mortality data for HIV-infected adults in the pre-treatment era from five African HIV cohort studies were combined to construct a life table, starting at age 15, smoothed with a Weibull model. Assuming that ART should be made available to anyone expected to die within 3 years, conditional 3-year survival probabilities were computed to represent proportions needing ART. The build-up of ART need in a successful programme continuously recruiting infected adults into treatment as they age to within 3 years of expected death was represented by annually extending the conditional survival range.

**Results:**

The Weibull model: survival probability in the infected state from age 15 = exp(−0.0073 × (age − 15)^1.69^) fitted the pooled age-specific mortality data very closely. Initial treatment need for infected persons increased rapidly with age, from 15% at age 20–24 to 32% at age 40–44 and 42% at age 60–64. Overall need in the treatment of naïve population was 24%, doubling within 5 years in a programme continually recruiting patients entering the high-risk period for dying.

**Conclusion:**

A reasonable projection of treatment need in an ART naive population can be made based on the age and gender profile of HIV-infected people.

## Introduction

The large number of people living with HIV/AIDS (PLWHA) in Africa places a huge burden on anti-retroviral therapy (ART) services. There is a need for simple tools to estimate treatment need amongst those who do not have access to ART services, and to gauge whether established services keep up with local treatment needs. Such estimates have been made at a national level using complex projection methods, such as the Spectrum package developed by UNAIDS (Stover *et al.* 2006; Mahy *et al.* 2010), which estimates the theoretical need for ART by projecting distributions of expected CD4 counts from an assumed pattern of past HIV incidence in the population. These estimates of national need can be compared with estimates of patients receiving treatment obtained from aggregated data from the national treatment programme.

National estimates of ART need are difficult to disaggregate for regional or district populations, as estimates of past incidence are not available at a local level, and because local prevalence patterns owe as much to different migratory movements by infected and uninfected persons as to the past incidence history of the locality. We propose a new approach, whereby the need for ART in HIV positive persons is estimated from the proportion that would be expected to die within a fixed number of years in the absence of treatment. This new method calculates ART needs specific for gender and age group, taking account of longer exposure and faster disease progression observed in older people, and cumulates estimated treatment need arising from improved survival of those accessing treatment.

The new approach could be applied in any sub-national population where HIV prevalence estimates are available from nationally representative population-based surveys [like the Demographic and Health (DHS) and AIDS Indicator Surveys (AIS)] that anonymously collect bio-markers of HIV status. It does not require further specialist information (such as CD4 count or viral load distributions) to estimate disease stage at a population level. This new method should therefore prove particularly useful for obtaining rough estimates of treatment need in populations where large proportions have never had HIV counselling and testing (HCT). It could also be used to estimate residual treatment need in population sub-groups that have not yet had any contact with care and treatment programmes, provided an approximate estimate of their age pattern of HIV prevalence can be made.

The raw data from which the age-specific mortality rates of untreated HIV positive persons were calculated was collected by the ALPHA network study centres (Maher *et al.* 2010) prior to ART availability. These mortality patterns were previously reported (Todd *et al.* 2007; Zaba *et al.* 2007), but the pooled data set has since been augmented with more pre-treatment era data, and improved analysis methods have been devised to construct age-specific mortality models for the HIV infected.

## Methods

In this analysis, we used data from five sites: Karonga (Malawi), Kisesa (NIMR, Tanzania), Umkhanykade (the Africa Centre for Health and Population Studies), Masaka (MRC/UVRI, Uganda) and Manicaland (BRTI, Zimbabwe). Table 1 presents some background characteristics of the sites contributing data. Further details about each site are given in the companion papers in this supplement.

**Table 1:** Background characteristics of study sites contributing data to pooled analysis

Study site	Location	Start of HIV (and demographic) surveillance	Start of ART availability	Population size, 2008	2008 HIV prevalence (%)
Karonga	Northern Malawi	2002	2004	32 000	7
Umkhanyakude	KwaZulu Natal, S. Africa	2003 (2000)	2004	86 000	22
Kisesa	North West Tanzania	1994	2005	28 000	7
Kyambaliwa	Southern Uganda	1989	2003	7 000	6
Manicaland	Eastern Zimbabwe	1998	2006*	30 000	16

ART, anti-retroviral therapy.

* Manicaland data were provided for the pooled data set up to 2005.

### Deaths and mortality rates of HIV infected

To estimate mortality rates among HIV-infected persons in the pre-treatment era, we used information on deaths occurring in the study areas to residents who were known to be HIV positive as a result of prior testing. The person-years at risk corresponding to these deaths were calculated from observed time since sero-conversion for incident cases (assumed to be half-way between last negative and first positive test), and from time since first positive test for prevalent cases. Periods during which infected persons were not under observation in the study were censored: left censored at age 15 or first move into study area; right censored at loss to follow-up or last interview; and interval censored if they left the study area and took up residence elsewhere, but then returned. HIV mortality in the absence of ART was calculated for periods up until ART became available in the study area (taken as 1 January 2004 in Karonga, Umkhanyakude and Kyambaliwa; 1 January 2005 in Kisesa, and 1 July 2006 in Manicaland). The methods for calculating mortality rates have been fully described previously (Todd *et al.* 2007; Zaba *et al.* 2007).

To calculate numbers in need of treatment at any time point, we also took account of deaths of residents who had never had an HIV test, or who had previously tested negative, but were diagnosed as AIDS deaths following a verbal autopsy (VA) interview with a family member. For AIDS deaths diagnosed at VA, subjects were assumed to have been infected for 10 years prior to death, or from mid-way between last negative test and death, or from date of entry into observation in the study, whichever of these dates was later.

Using the pooled data set, an age-based Kaplan–Meier survival function was constructed to represent mortality experience of HIV positive adults, with an origin at age 15. No upper age limit was imposed on the data, but observations after age 75 are sparse, and so, resulting graphs and tables are truncated at this age. Non-parametric tests (log rank and Cox proportional hazards diagnostics) were performed on the survival data to investigate whether there were significant differences in mortality levels or age patterns between study sites and by gender (allowing for clustering between sites).

### Model life-table fitting

Parametric regression techniques as implemented in Stata version 11 (StataCorp, 2009) were used to fit Weibull models to represent age-specific mortality among the HIV infected in the pre-treatment era. This two parameter model is characterised by λ, the overall mortality level and ϕ, the pattern of increase of mortality with age, to define *P*(*a*), the probability of surviving in the infected state from age 15 to age *a*


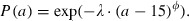
(1)

The probability of dying in the next *n* years for an HIV-infected person aged *a* in a population where ART is not yet available (i.e. available for 0 years) is thus given by *D*(*a*, *n*, 0)


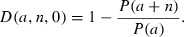
(2)

### Numbers needing treatment

Assuming that the proportion needing ART can be equated to the proportion expected to die in the next *n* years in the absence of ART, this expression provides a way of estimating treatment need in a population where ART has not been previously available: *n* can be thought of as the mean time from optimal treatment start to expected AIDS death. In this article, a default value of *n* = 3 years has been chosen – to put this choice into perspective and compare with more complex models, Table 2 shows the range of assumptions used in current and past versions of the SPECTRUM model, relating mean or median time from estimated CD4 thresholds to death.

**Table 2:** CD4 Estimated median and mean time from CD4 treatment threshold to death

CD4 treatment initiation threshold	Median time to death in years {figures in braces are author’s estimates for both sexes based on 67% infected female}	Mean time to death in years {figures in braces are author’s estimates based on median using Poisson approximation}	Source
200	1.5 males, 2.0 females {1.83 both sexes}	{2.6}	Mahy *et al.* (2010)
200	2.1	{3.0}	Stover *et al.* (2006)
200	2.7 (credibility limits 0.8–8.4)	5.2 (credibility limits 1.5–15.9)	Wandel *et al.* (2008)
275	5.0 (credibility limits 1.6–13.8)	7.4 (credibility limits 2.5–20.5	Wandel *et al.* (2008)
350	3.6 males, 3.9 females {3.8 both sexes}	{5.5}	Mahy *et al.* (2010)
350	4.0	{5.7}	Stover *et al.* (2006)
350	7.6 (credibility limits 3.0–18.3)	9.8 (credibility limits 3.8–23.7)	Wandel *et al.* (2008)

Table 2 shows that most expositions quote only median time (not mean time) from CD4 threshold to death; that the range of estimated values of mean time to death for commonly used CD4 thresholds (200 and 350) are very wide (2.6–5.2 years and 5.5–9.8 years respectively) and that where credibility limits have been estimated these are also very wide – up to 30% and 300% of the mean value (Wandel *et al.* 2008). We suggest that our default value of *n* = 3 be regarded as notionally equivalent to a low CD4 threshold of around 200 (as used at the start of most national ART programmes), whereas the currently proposed CD4 threshold of 350 can be regarded as notionally equivalent to a value of *n* = 6.

Anti-retroviral therapy programmes that recruit everyone who is expected to die in the next *n* years in the absence of treatment will need to recruit additional people in every calendar year as infected cohorts age and more of them enter into the high death risk period: *y* years following the start of the programme, the proportion of HIV-infected people currently aged *a* who will need treatment is *D*(*a*, *n*, *y*) where


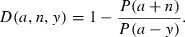
(3)

Strictly speaking, this represents the proportion of infected people aged *a − y* in year 0 who are expected to need treatment in *y* year’s time. Applying this to infected persons aged *a* in year *y* is equivalent to assuming that the mortality of those on treatment can be brought down, on average, to the same level as the mortality of those HIV-infected persons originally classified as not yet in need of treatment.

To calculate the actual number needing treatment, these proportions are applied to the numerical age distribution *H*(*a, y*) of HIV positive persons identified in the population in a particular year, allowing for the number of years ***y*** that have passed since the start of ART availability. An adjustment can be made for persons of unknown HIV status by assuming that prevalence amongst those of unknown status is the same as among those whose status is known in each age group. The overall requirements for ART, and progress of the programme in meeting ART needs can be judged by summing the numbers across age groups, although it may also be useful to monitor trends by gender and broad age groups to judge which population sub-groups are less well served. The total number *T*(*y*) needing treatment in year y is given by:


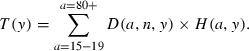
(4)

Equation 4 estimates the total ART need *y* years after the start of the programme, in a programme that operates at maximum efficiency in recruiting each year all the new patients in need of therapy. Programmes that fail to recruit all those defined as in need in earlier years will not ‘need’ to recruit the full quota of patients in later years, because some of those who should have been recruited from the high mortality risk group will have died in the intervening years, so that the proportion in need in later years will be reduced. We suggest the use of linear interpolation for estimating *R*(*y* + 1), the reduced treatment need in the year *y* + 1 based on the actual proportion receiving treatment in year *y*, *R*(*y*).

Suppose the actual proportion treated in year *y*, *R*(*y*), is related to the maximum theoretical need *T*(*z*) in an earlier year *z*, according to *T*(*z*) ≤ *R*(*y*) ≤ *T*(*z* + 1) where *y* ≥ *z*. The linear interpolation estimate for the reduced need in year *y* + 1 is given by:


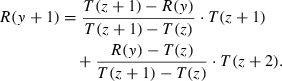
(5)

## Results

### Mortality patterns in HIV infected

A summary of the basic data on mortality of HIV-infected persons contributed by each study site is shown in Table 3. Umkhanyakude in South Africa contributed the largest number of observations and along with Karonga in Malawi (which contributed the fewest observations) experienced higher crude death rates; Kisesa and Manicaland experienced lower death rates amongst the HIV infected.

**Table 3:** Deaths, person-years of observation and crude death rates in HIV positive population in five ALPHA network sites, pre-ART roll-out

Study site	Number of deaths of HIV infected	Person-years observation of HIV infected (thousands)	Crude death rate among HIV infected (per thousand)	95% confidence intervals	
Karonga	70	0.663	105.5	83.5	133.4
Umkhanyakude	1865	19.311	96.6	92.3	101.1
Kisesa	322	4.389	73.4	65.8	81.8
Kyambaliwa	534	6.157	86.7	79.7	94.4
Manicaland	495	6.736	73.5	67.3	80.3
Total	3286	37.255	88.2	85.2	91.3

ART, anti-retroviral therapy.

The inter-site differences are fairly small and insignificant compared with other mortality differentials – e.g. by sex: the crude death rate for HIV-infected men pre-ART was 110.2 per thousand (104.7–116.0), for women 75.9 (72.5–79.5); by infection status: uninfected 5.1 (4.8–5.4), unknown status 21.5 (20.8–22.4). In the 3 years following the introduction of ART, the crude death rate for HIV-infected persons across all sites fell from 88.2 (85.2–91.3) to 64.3 per thousand (61.9–66.9).

**Figure 1:**
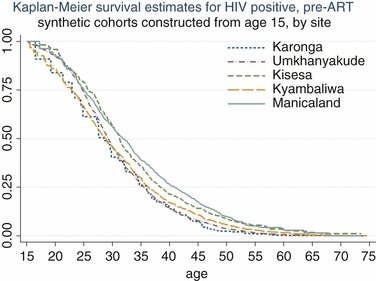
Survival from age 15 for HIV-infected persons in five ALPHA network sites, pre-anti-retroviral therapy roll-out.

Differences in crude death rates may be due to the age structure of the infected populations, as mortality amongst the HIV infected rises rapidly with age, and the age structure of the HIV infected varies according to the maturity of the epidemic and the overall age structure of the population. It is therefore appropriate to compare age-specific survival patterns, as in Figure 1, which shows age-based Kaplan–Meier functions in the HIV-infected study populations based on pre-ART mortality.

Non-parametric Cox regression results suggested that the differences between sites were not important, once the age structure of the infected population in the different sites was accounted for. Between ages 25 and 50, where 80% of AIDS deaths occur, compared with Karonga, hazard ratios were Umkhanyakude 1.22 (0.92–1.63), Kisesa 0.99 (0.73–1.34), Kyambuliwa 1.07 (0.80–1.44) and Manicaland 0.83 (0.62–1.11), and proportional hazard assumptions were satisfied (chi squared statistic test on Schoenfeld residuals with four degrees of freedom is 4.41, *P* = 0.3530).

**Figure 2:**
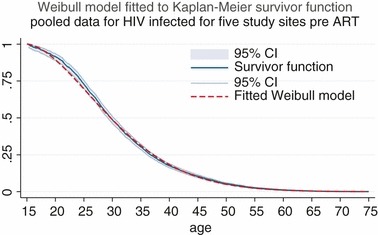
Observed survival pattern for HIV positive pre-anti-retroviral therapy in pooled data set, with fitted Weibull model.

A Weibull parametric model was fitted to the pooled mortality data for both sexes to obtain a mathematical representation of conditional survival of HIV-infected persons by age –Figure 2 shows that this model is an excellent fit to the observed data (*r*^2^ = 0.9992 comparing Kaplan–Meier and Weibull at all ages for which deaths were reported). The parameters for the best fitting regression model were λ = 0.0073 (95% CI: 0.0059–0.0091) and ϕ = 1.69 (95% CI: 1.59–1.79). The curve representing the model fitting is obtained by substituting these values in Eqn 1.

### ART treatment need by age and programme year

The mortality pattern described by the Weibull model was used to predict the proportions expected to die within 3 years in a treatment naive population, using Eqn 2 with *n* = 3. The results are shown in the first column of Table 2, averaged over 5 year age groups. The remaining columns show the maximum theoretical treatment need during each of the 5 years following the start of the programme. The entries show the proportion of currently infected persons needing treatment in each age group, obtained by applying Eqn 3 with *n* = 3, and *y* as indicated by the column heading. At the start of the programme, the proportion of HIV-infected persons needing treatment in the 20–24, 40–44 and 60–64 age groups would be 15%, 32% and 40%, respectively, and this would rise to 30%, 61% and 75% of the currently infected in the respective age groups, after 5 years of the programme.

The last three rows show the overall proportion of infected persons needing treatment when the age-specific proportions are applied to the average age distributions of infected persons, by gender, in the pooled data contributed by sites since start of surveillance. As the need for treatment escalates rapidly with age, the overall proportion of infected women needing treatment is lower than for men, because the age distribution of infected women is younger. A treatment programme that succeeded in restoring those otherwise facing death in the next 3 years to the same level of mortality experienced by those outside the high risk group, and which continued to recruit onto treatment all those entering the high risk group over the coming years would need to grow rapidly, almost doubling in size within 5 years.

We can explore the scale and trend of ART needs in programmes with different theoretical recruitment strategies using different values of *n* (the time from treatment start to expected AIDS death) in Eqns 2 and 3. The bottom line of Table 4 shows that the overall starting need for ART in a programme that aimed to recruit those expected to die within 3 years would be 24%, almost doubling to 46.5% in 5 years in a population whose HIV prevalence pattern by age was the average of that observed in the ALPHA sites. If a less ambitious programme only aimed to recruit those who were expected to die within a year, the overall proportion needing treatment at the start of the programme would be about 8.5%, whereas a more ambitious programme, aiming to recruit those expected to die within 6 years would categorise 43.5% of the HIV-infected population as in need of treatment at baseline. However, a programme aiming to recruit only those within a year of death would have to grow very fast: within 5 years it would need to quadruple in size, reaching 36.5%, to keep up with this modest aim, whereas a successful programme aiming to treat those 6 years from death from the very start would only need to grow by 40% to reach 59% coverage of all infected persons over the course of 5 years.

**Table 4:** Model proportion needing treatment by age group and programme year, defining treatment need as proportion expected to die within 3 years in the absence of treatment

	Years since start of treatment programme					
	
Current age group	0	1	2	3	4	5
15–19	0.091	0.112	0.124	0.131	0.134	0.135
20–24	0.153	0.196	0.230	0.258	0.282	0.300
25–29	0.203	0.259	0.306	0.347	0.383	0.414
30–34	0.245	0.309	0.364	0.413	0.456	0.493
35–39	0.282	0.353	0.415	0.469	0.517	0.558
40–44	0.315	0.392	0.459	0.517	0.568	0.612
45–49	0.344	0.427	0.498	0.559	0.611	0.656
50–54	0.371	0.459	0.533	0.595	0.648	0.694
55–59	0.396	0.487	0.563	0.627	0.680	0.725
60–64	0.419	0.513	0.591	0.655	0.709	0.753
65–69	0.441	0.538	0.616	0.681	0.734	0.777
70–74	0.461	0.560	0.639	0.704	0.756	0.799
75+	0.496	0.602	0.682	0.745	0.795	0.835
All age proportion given average HIV prevalence by age in ALPHA sites						
Males	0.262	0.327	0.382	0.430	0.472	0.508
Females	0.228	0.284	0.333	0.374	0.410	0.441
Both sexes	0.240	0.299	0.350	0.394	0.432	0.465

### Estimating ART need in Tanzania, 2007–2010: a worked example

An illustrative application of the proposed method to estimate the theoretical need for ART in Tanzania between 2007 and 2010 is shown in Table 5. The basic input data are taken from the 2007 DHS survey of Tanzania (DHS, 2011), which took place 3 years after the start of Tanzania’s national ART programme, launched in 2004. The mid-year population estimate for Tanzania in 2007 is based on the UN population division medium estimate (http://esa.un.org/wpp/unpp/panel_population.htm; accessed 4 January 2012). For ages 15–49, age-specific HIV prevalence estimates were taken from the DHS. We assumed that prevalence at older ages declined by 12% for each 5-year age group, but then pro-rated prevalence in all these age groups to ensure that HIV prevalence in the whole 50+ age group was lower than prevalence at ages 15–49 by a factor of 1.7 – this overall age relationship is based on observed prevalence in the Kisesa cohort study (Mwita *et al.* 2007) in Northern Tanzania, and is used by the National AIDS Control Programme as a standard estimate for HIV prevalence at ages 50 + in the decade 2000–2010 (Ministry of Health, 2011). This effectively implies zero HIV prevalence at ages 80 and over.

**Table 5:** ART need estimates for Tanzania, 2007–2010, based on HIV prevalence reported in 2007 DHS survey

Tanzania 2007 – DHS survey					Number needing treatment *N* years after programme start			
	
	Age distribution (%)	Estimated number (thousands)	HIV prevalence (%)	Estimated number HIV infected (thousands)	0 (thousands)	1 (thousands)	2 (thousands)	3 (thousands)
								
Age group	a	b	c	d	e	f	g	h
<5	18.1	7452						
5–9	15.5	6382						
10–14	13.6	5600						
15–19	9.7	3994	1.0	40	4	4	5	5
20–24	7.4	3047	4.3	131	20	25	30	33
25–29	6.8	2800	6.7	188	38	48	57	64
30–34	6.0	2470	9.1	225	55	69	82	93
35–39	5.1	2100	10.0	210	59	74	87	99
40–44	3.5	1441	7.2	104	33	41	48	54
45–49	3.3	1359	6.4	87	30	37	43	49
50–54	2.5	1029	5.8^*^	60	22	27	32	36
55–59	2.3	947	4.5^*^	43	17	21	24	27
60–64	1.7	700	3.0^*^	21	9	11	12	14
65–69	1.4	576	1.6^*^	9	4	5	6	6
70–74	1.2	494	0.7^*^	3	2	2	2	2
75–79	0.8	329	0.2^*^	1	0	0	0	0
80+	1.0	412	0.0^*^	0	0	0	0	0
Missing	0.1							
Total	100	41 132		1121	293	365	428	482
Need ART as % of HIV infected in 2007					26%	33%	38%	43%

ART, anti-retroviral therapy.

a: from 2007 Tanzania DHS (DHS, 2011), table 2.1, page 9; b: 41 132 = population total for 2007 from UN medium projection, distributed according to column a; c: from 2007 Tanzania DHS (DHS, 2011), table 9.3, page 138; d: b × c; e, f, g, h: corresponding columns of table 2 multiplied by column d (DHS, 2011); ^*^Extended to ages 50+ on the basis of scaled Kisesa data.

Applying the model to the estimated 1.121 million HIV-infected adults in Tanzania suggests that if 2007 had been the initial year of the ART programme with the aim of reaching those infected persons expected to die within 3 years, the number needing treatment would be 293 000. Based on a CD4 threshold of 200 for treatment initiation, the SPECTRUM model estimate for treatment need in 2007 for Tanzania is 279 000 (J. Stover, personal communication ), about 5% lower than the estimate based on this simple model.

Since 2007 was actually year 3 of the programme, our model indicates that a theoretical target of a totally successful programme would be 482 000. The reported cumulative number of patients recruited to the treatment programme by 2007 was in fact 164 000, much lower than any of these theoretical targets (Ministry of Health, 2011).

We can use Eqn 5 to investigate whether the programme is catching up on targets in subsequent years. Putting *T*(*z*) = 0, *T*(*z* + 1) = 293, *T*(*z* + 2) = 365, *T*(*z* + 3) = 428 and given *R*(*y*) = 164, the reduced needs in 2008 and 2009 would be given by:


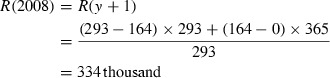



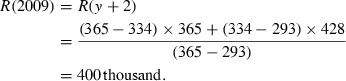


The reported cumulated number recruited to the Tanzanian ART programme by 2008 was 283 000 (Ministry of Health, 2011), so by 2008, the programme did not manage to catch up even with the reduced target number implied by this model. However, by 2009, the programme had managed to recruit a cumulated total of 456 000 (Ministry of Health, 2011), exceeding the reduced target number of 400 000 for 2009. The SPECTRUM estimates of ART need in 2008 and 2009, respectively, were 290 000 and 316 000, which would seem rather low, given actual programme recruitment, although the official recruitment figures do not indicate the number currently receiving treatment.

### Estimating ART need in the ALPHA study sites

Table 6 shows the results of applying this model to the actual age distribution of the infected population in each study site, by gender, to estimate the minimum initial need in 2004, close to the start year for most national programmes, and the maximum theoretical need in 2008, if programmes had successfully recruited all those expected to die within 3 years. Estimates are not available for Manicaland for 2008, as data from that site were only contributed to the pooled data set up to 2005. The overall proportions needing treatment are higher in the actual surveillance populations than in the ‘all age’ row in Table 4, because the average age distribution of infected persons in the pooled data set in the pre-ART era was younger than the distribution of infected persons in the surveillance sites in 2004 and 2008. In all sites for both years, the proportion of HIV-infected males needing treatment is higher than the proportion of females needing treatment, reflecting the older age distribution and higher mortality of the men. Karonga and Kyambuliwa have a slightly higher overall need than the other sites. The actual proportions on treatment in each site are discussed in papers elsewhere in this volume (Alison *et al.* 2012).

**Table 6:** Model predictions of the proportions of HIV infected and overall number needing Ante-Retroviral Treatment in selected calendar years in five ALPHA network study sites

	Predicted proportion (and number) of HIV infected needing treatment				Ratio of treatment need in 2008–2004	
		
	Year					
			
	2004		2008			
				
Site	Males	Females	Males	Females	Proportion	Number
Karonga	0.285 (157)	0.245 (211)	0.525 (308)	0.472 (434)	1.9	2.0
Umkhanyakude	0.260 (857)	0.230 (1590)	0.489 (1296)	0.441 (2855)	1.9	1.7
Kisesa	0.260 (101)	0.234 (115)	0.464 (132)	0.438 (180)	1.8	1.5
Kyambaliwa	0.276 (74)	0.241 (91)	0.521 (149)	0.458 (204)	1.9	2.1
Manicaland	0.277 (75)	0.264 (150)				

**Figure 3:**
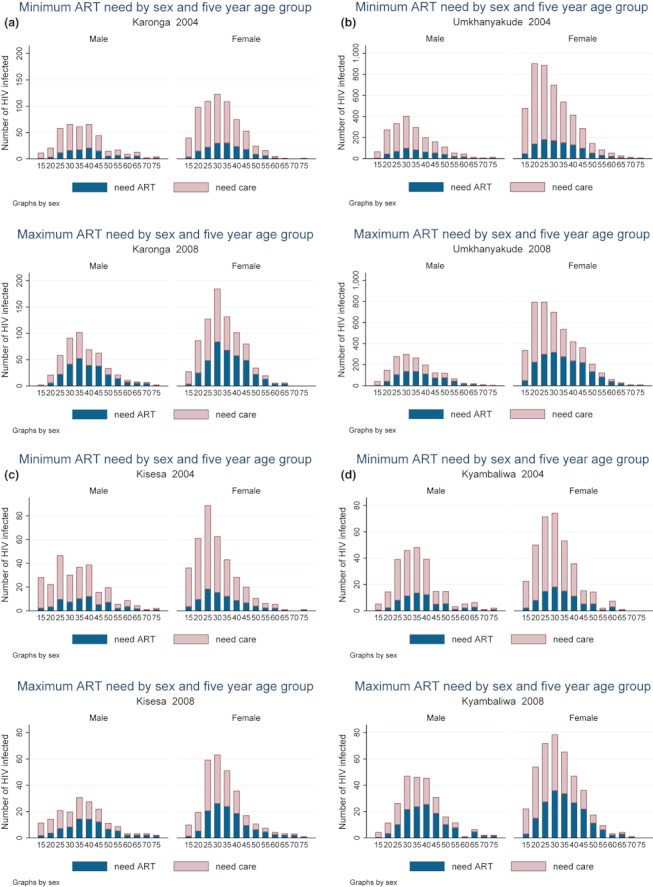
Number of HIV positive persons needing treatment or needing care by study site and gender at start of anti-retroviral therapy availability in 2004 and in growing programmes by 2008.

Figure 3 shows information about the age–gender distribution of people needing treatment in four of the sites in 2004 and in 2008. The years were chosen to contrast the situation at the start of the programmes in populations that were treatment naive, and 4 years later when successful programmes should have built on earlier achievements. The proportions from the model presented in Table 4 are applied to the actual numbers of HIV positive persons observed in the cohort at the start of these years, assuming that prevalence in residents of unknown status is the same as those whose status has been measured. The number in need of ART treatment is shown by the dark parts of the bars, and the total height of the stacked bar represents the number of HIV infected, so that the pale part of the stacked bar represents the rest of the HIV-infected people in each age group, who are not classified as needing treatment, but who should be receiving monitoring care with regular checks on CD4 counts and disease stage.

Comparing the age-specific and total numbers across the study sites, the following features emerge: overall, more women than men need treatment, as there are higher numbers of infected women. The sex ratio F:M among those needing treatment ranges from around 1.2 (in Kisesa, Karonga and Kyambuliwa) to 1.9 (in Manicaland and Umkhanyakude) and rises slightly over time. However, the excess in the number of women needing care rather than treatment is even larger than the corresponding number for men (sex ratios range from 1.5 in East African sites to 3.2 in Southern African sites), as lower proportions of women need treatment because their age distributions are younger. There is a clear peak age for treatment need in women at age 30–34 in all sites, whereas for men, treatment need is more evenly spread across the age groups, with a slightly older peak spread over ages 35–44.

Growth in numbers of people needing treatment differs from growth in the proportions needing treatment because of natural and migration-driven changes in the study populations. In Karonga and Kyambuliwa numbers, needing treatment increase faster than proportions because of increases in total numbers infected of the order of 10% over the 4-year period, and in Karonga, this increase is in part fuelled by the overall growth of the population in the study site.

## Discussion

The method provides a simple alternative to epidemic projection models for estimating age-specific ART need in populations in any year that age-specific HIV prevalence is known or has been estimated. It uses age-specific mortality patterns recorded for HIV-infected persons in the era before widespread availability of ART to determine the proportion of HIV-infected persons who are expected to die within a specified number of years – this proportion represents the deaths that it is hoped to avert by enrolling people on the ART programme. The method does not require knowledge of HIV incidence rates in the population, and can be applied in sub-national populations where prevalence may be determined by local migration patterns as well as by incidence rates. It is well known that the rate of progression from infection to death increases steadily with age (Todd *et al.* 2007) – this method alerts us to the corresponding importance of age as a determinant of ART need, a factor that tends to be overlooked if it is assumed that a fixed proportion of treatment naive HIV-infected people have CD4 counts low enough to qualify for ART.

This estimation technique can be applied in populations with large numbers of people already on treatment, who are included in the HIV prevalence estimate. The outputs represent those who should be currently receiving HIV treatment, whereas some national programmes only report cumulative numbers who have ever received ART. It is assumed that anyone already receiving treatment belongs to the treatment need group defined on the basis of mortality expectations, and this simple model has not yet been refined to handle changes over time in the criteria for admission of people to the ART programme.

The age-specific mortality schedule used in this article is based on all cause mortality of the HIV infected in the pre-treatment era. Had we adjusted for non-AIDS deaths among the HIV infected, our estimates of the proportion needing treatment (expected to die in the next 3 years) would have been approximately 2% lower at ages 15–29 and 5% lower at ages 50+, based on ‘net’ mortality patterns described previously in these populations (Marston *et al.* 2007).

Another important assumption concerns the approximate equality in mortality between infected persons already on treatment and those judged as not yet needing treatment. This assumption can be looked at in terms of long-term goals of a treatment programme – if the mortality of those classified as not yet needing treatment is appreciably higher than the mortality of those on treatment, this suggests that too many high risk persons are left in the group designated as not yet in need of treatment, setting a more inclusive target for acceptance onto treatment would lower mortality among the group designated as not yet needing treatment. Conversely, if mortality is much higher amongst those on treatment, this suggests that the available treatment is not effective enough to warrant the inclusion of such a large fraction, as there is no overall mortality gain from their inclusion.

As evidence from ART clinics suggests that mortality may be very high in the first year following treatment initiation, it will be important to measure mortality of the two groups over a period of time commensurate with the mortality prevention goals of the selection criteria – e.g. if the CD4 count threshold for recruitment onto treatment is supposed to be equivalent to preventing expected AIDS deaths in the next 3 years, then the programme would have to have recruited a proportion of infected people equivalent to theoretical need at baseline + 3 years (about 35%) to ensure a balance of long- and short-term ART users in whom mortality effects of treatment could be reasonably assessed. One reason that we might expect high mortality for those on ART is that patients who fail ART may not be moved quickly to a secondary treatment regime, and remain on failing regimens for prolonged periods that would expose them to a substantially increased risk of death. Programmes with high levels of treatment discontinuation would also end up with higher mortality amongst the ever-treated than is assumed in this method.

If the long-term mortality of those receiving ART treatment turns out to be lower than mortality of those not yet needing treatment, this method would consistently under-estimate future treatment need, if mortality of those on treatment turns out to be higher than mortality of those classified as not yet needing treatment future treatment needs will have been over-estimated.

In small geographically defined populations, prevalence is determined as much by movement of infected people into and out of the region as by incidence amongst the uninfected and deaths of infected persons (Mwita *et al.* 2007). In this situation, the methods presented here will be valid, provided that migration is not related to treatment seeking – i.e. provided that in-migration is not an influx of people seeking treatment in the locality or that out-migration is not motivated by the necessity to seek treatment outside the locality. Where prevalence estimates are themselves subject to uncertainty – e.g. due to missing data as a result of test refusals, estimates of ART need will also be affected (Bärnighausen *et al.* 2011).

We have illustrated the estimation procedures above in the same HIV cohort studies that supplied the data for calculating the age-specific mortality rates for those infected with HIV. Some of these studies have now achieved very high levels of HCT, and have representative data on CD4 counts in the general population, i.e. direct measures of ART need (Malaza *et al.* 2011). However, the real power of the method will lie in its application in national and sub-national populations where HCT service use is far from universal, but where surveillance data are available – such as anonymous HIV testing in the context of a nationally representative survey like the DHS (DHS, 2011) – and can be used to obtain an age distribution of HIV-infected persons.

One advantage of the method is that if alternative methods are developed to select people for treatment – e.g. relying on viral load or some new type of clinical observation, rather than CD4 count – these could also be described in terms of years of AIDS-related mortality that we seek to avert. The simple method allows a health planner to envisage the size of a local ART programme that would be required, given a desired impact in terms of average years of life gained by HIV-infected persons.
